# Supplementary material for: Low Efficacy of Genetic Tests for the Diagnosis of Primary Lymphedema Prompts Novel Insights into the Underlying Molecular Pathways
Source: Int J Mol Sci. 2022 Jul 3;23(13):7414. doi: 10.3390/ijms23137414 (PMC9267137; doi:10.3390/ijms23137414)
Supplement: Supplementary file 1 [file ijms-23-07414-s001.zip › ijms-1740920-supplementary.pdf]

— Supplementary Material —

Low efficacy of genetic tests for the diagnosis of primary lymphedema prompts novel insights into the underlying molecular pathways

Gabriele Bonetti [1,*], Stefano Paolacci [1], Michele Samaja [2], Paolo Enrico Maltese [1], Sandro Michelini [3], Serena Michelini [4], Silvia Michelini [5], Maurizio Ricci [6], Marina Cestari [7,8], Astrit Dautaj [1], Maria Chiara Medori [1], Matteo Bertelli [1,2,9]

1 MAGI's LAB, Rovereto 38068, Italy; stefano.paolacci@assomagi.org (S.P.); paolo.maltese@assomagi.org (P.E.M.); astrit.dautaj@assomagi.org (A.D.); chiara.medori@assomagi.org (M.C.M.); matteo.bertelli@assomagi.org (M.B.)
2 MAGI Group, San Felice del Benaco 25010, Italy; michele.samaja@assomagi.org
3 Vascular Diagnostics and Rehabilitation Service, Marino Hospital, ASL Roma 6, Marino 00047, Italy; sandro.michelini@aslroma6.it
4 Unit of Physical Medicine, "Sapienza" University of Rome, Rome 00185, Italy; serenamichelini@gmail.com
5 Neurosurgery, University of Tor Vergata, Rome 00133, Italy; silviamichelini1992@gmail.com
6 Division of Rehabilitation Medicine, Azienda Ospedaliero-Universitaria, Ospedali Riuniti di Ancona, Ancona 60126, Italy; maurizio.ricci@ospedaliriuniti.marche.it
7 Study Centre Pianeta Linfedema, Terni 05100, Italy; cestari.marina@libero.it
8 Lymphology Sector of the Rehabilitation Service, USLUmbria2, Terni 05100, Italy; cestari.marina@libero.it
9 MAGI Euregio, Bolzano 39100, Italy

* Correspondence: gabriele.bonetti@assomagi.org; Tel.: +39-0365-62-061

List of diagnostic and candidate genes

Table S1. List of diagnostic genes for primary lymphedema. Name of the gene, OMIM number, gene-phenotype relationship, relative reference and inheritance, involved pathway, relative reference and effect on the pathway are reported. References are from OMIM, Kegg, or literature.

Gene	OMIM	Gene-Phenotype relationship	Reference	Inheritance	Pathways	Reference	Effect
**ADAMTS3**	605011	Hennekam lymphangiectasia-lymphedema syndrome 3	618154	AR	VEGFR3 pathway	[24]	Activation
**AKT1**	164730	Proteus syndrome, somatic	176920		Ras pathway	map04014	Inactivation
					PI3K/AKT pathway	map04151	Activation
**BRAF**	164757	Noonan syndrome 7	613706	AD	MAPK pathway	map04010	Activation
**CBL**	165360	Noonan syndrome-like disorder with or without juvenile myelomonocytic leukemia	613563	AD	Ras pathway	[116]	Inactivation
**CCBE1**	612753	Hennekam lymphangiectasia-lymphedema syndrome 1	235510	AR	VEGFR3 pathway	[21]	Activation
**CELSR1**	604523	Lymphatic malformation 9	619319	AD	Planar cell polarity	[117]	Activation
**EPHB4**	600011	Capillary malformation-arteriovenous malformation 2	618196	AD	Ras pathway	[118]	Inactivation
		Lymphatic malformation 7	617300	AD			
**FAT4**	612411	Hennekam lymphangiectasia-lymphedema syndrome 2	616006	AR	Planar cell polarity	[89]	Activation
**FLT4**	136352	Lymphatic malformation 1	153100	AD	Ras pathway	map04014	Activation
					PI3K/AKT pathway	map04151	Activation

**FOXC2**	602402	Lymphedema-distichiasis syndrome	153400	AD	Transcription factors	[75]	Activation
**GATA2**	137295	Emberger syndrome	614038	AD	Transcription factors	[12]	Activation
**GJA1**	121014	Oculodentodigital dysplasia	164200		Lymphatic valve development	[91]	Activation
**GJC2**	608803	Lymphatic malformation 3	613480	AD	Lymphatic valve development	[92]	Activation
**HGF**	142409	Primary lymphedema	[8]		Ras pathway	map04014	Activation
					PI3K/AKT pathway	map04151	Activation
**HRAS**	190020	Costello syndrome	218040	AD	Ras pathway	map04014	Activation
					PI3K/AKT pathway	map04151	Activation
**KIF11**	148760	Microcephaly with or without chorioretinopathy, lymphedema, or mental retardation	152950	AD	PI3K/AKT pathway	[119]	Activation
**KRAS**	190070	Noonan syndrome 3	609942	AD	Ras pathway	map04014	Activation
					PI3K/AKT pathway	map04151	Activation
**NRAS**	164790	Noonan syndrome 6	613224	AD	Ras pathway	map04014	Activation
					PI3K/AKT pathway	map04151	Activation
**PIEZO1**	611184	Lymphatic malformation 6	616843	AR	PI3K/AKT pathway	[120]	Activation
					Lymphatic valve development	[94]	Activation
**PIK3CA**	171834	CLAPO syndrome, somatic	613089		Ras pathway	map04014	Activation
		CLOVE syndrome, somatic	612918		PI3K/AKT pathway	map04151	Activation
**PTPN11**	176876	Noonan syndrome 1	163950	AD	Ras pathway	map04014	Activation
					PI3K/AKT pathway	[121]	Activation
**PTPN14**	603155	Choanal atresia and lymphedema	613611	AR	VEGFR3 pathway	[25]	Activation
**RASA1**	139150	Capillary malformation-arteriovenous malformation 1	608354	AD	Ras pathway	map04014	Inactivation
**RIT1**	609591	Noonan syndrome 8	615355	AD	Ras pathway	[50]	Activation
**SHOC2**	602775	Noonan syndrome-like with loose anagen hair 1	607721	AD	Ras pathway	map04014	Activation
**SOS1**	182530	Noonan syndrome 4	[122]	AD	Ras pathway	map04014	Activation
					PI3K/AKT pathway	map04151	Activation
**SOX18**	601618	Hypotrichosis-lymphedema-telangiectasia syndrome	607823	AR	Transcription factors	[12]	Activation
		Hypotrichosis-lymphedema-telangiectasia-renal defect syndrome	137940	AD			
**VEGFC**	601528	Lymphatic malformation 4	615907	AD	Ras pathway	map04014	Activation
					PI3K/AKT pathway	map04151	Activation

Table S2. List of candidate genes for primary lymphedema. Name of the gene, OMIM number, gene-phenotype relationship, relative reference and inheritance, involved pathway, relative reference and effect on the pathway are reported. References are from OMIM, Kegg, or literature.

Gene	OMIM	Gene-Phenotype relationship	Reference	Inheritance	Pathways	Reference	Effect
**ACKR2**	602648	Lymphatic vascular development	[105]		Macrophage recruitment/activation	[105]	Activation
**ADM**	103275	Lymphatic vascular development	[123]		Ras pathway	[124]	Activation
		Predisposition to secondary lymphedema	[125]		PI3K/AKT pathway	[124]	Activation
					Vascular smooth muscle contraction	map04270	Activation
**ANGPT2**	601922	Lymphatic malformation 10	619369	AD	Ras pathway	map04014	Inactivation
					PI3K/AKT pathway	map04151	Inactivation
**ANGPTL4**	605910	Lymphatic vascular development	[126]		Rho and cytoskeletal remodelling pathway	[127]	Activation
					PI3K/AKT pathway	[128]	Inactivation
**ARAF**	311010	Noonan syndrome	H01738		Ras pathway	map04010	Activation
		Noonan syndrome and related disorders	H00523		Vascular smooth muscle contraction	map04270	Activation
**ARAP3**	606647	Lymphatic vascular development	[129,130]		Rho and cytoskeletal remodelling pathway	map04015	Activation
					PI3K/AKT pathway	[130]	Activation
**PPP1R13B**	606455	Lymphatic vascular development	[131]		NFkB pathway	[132]	Inactivation
**CALCRL**	114190	?Lymphatic malformation 8	618773	AR	Ras pathway	[124]	Activation
					PI3K/AKT pathway	[124]	Activation
					Vascular smooth muscle contraction	map04270	Activation
**CDH5**	601120	Lymphedema	[133]		Rho and cytoskeletal remodelling pathway	[134]	Activation
					Mechanotrasduction	[99]	Activation
					PI3K/AKT pathway	[79]	Activation
					β-catenin pathway	[79]	Activation
**CDK5**	123831	Lymphatic vascular development	[78]		Transcription factors	[78]	Activation
**CYP26B1**	605207	Lymphedema	[102]		RA pathway	[102]	Activation
**DCHS1**	603057	Lymphatic valve development	[90]		Hippo pathway	map04392	Activation
**EFNB2**	600527	Lymphatic valve development	[135]		Lymphatic valve development	[135]	Activation
**EMILIN1**	130660	Lymphatic valve development	[136]		Lymphatic valve development	[136]	Activation
**FABP4**	600434	Lymphedema	[137]		Macrophage recruitment/activation	[106]	Activation
**FLT1**	165070	Lymphatic vascular development	[138]		Ras pathway	map04014	Activation
					PI3K/AKT pathway	map04151	Activation
**FOXC1**	601090	Lymphatic vascular development	[139]		Transcription factors	[140]	Activation
**FOXC2-AS1**		Lymphedema	[75]		Transcription factors	[75]	Activation
**GDF2**	605120	Lymphatic vascular development	[141]		Transcription factors	[82,142]	Inactivation
		Lymphatic valve development	[141]				
**GJA4**	121012	Lymphatic valve development	[142]		Lymphatic valve development	[142]	Activation
		Predisposition to secondary lymphedema	[143]				
**HHEX**	604420	Lymphatic vascular development	[144]		Transcription factors	[144]	Activation

**HOXD10**	142984	Lymphatic vascular development	[145]		Rho and cytoskeletal remodelling pathway	[145]	Inactivation
**IKBKG**	300248	Ectodermal dysplasia and immunodeficiency 1	300291	XLR	Ras pathway	map04014	Activation
					PI3K/AKT pathway	map04151	Activation
					NFkB pathway	[146]	Activation
**ITGA5**	135620	Lymphatic vascular development	[147]		PI3K/AKT pathway	map04151	Activation
**ITGA9**	603963	Lymphatic valve development	[148]		PI3K/AKT pathway	map04151	Activation
**LCP2**	601603	Predisposition to secondary lymphedema	[149]		Rho and cytoskeletal remodelling pathway	[150]	Activation
**LPAR1**	602282	Lymphedema	[151]		PI3K/AKT pathway	map04151	Activation
**LPAR2**	605110	Lymphedema	[151]		PI3K/AKT pathway	map04151	Activation
**LPAR3**	605106	Lymphatic vascular development	[152]		Ras pathway	[153]	Activation
		Lymphatic vascular development	[152]		PI3K/AKT pathway	map04151	Activation
**LPAR4**	300086	Lymphatic vascular development	[153]		PI3K/AKT pathway	map04151	Activation
**LYVE1**	605702	Lymphedema	[154]		PI3K/AKT pathway	map04151	Activation
**LZTR1**	600574	Noonan syndrome 10	616564	AD	Ras pathway	[155]	Inactivation
		Noonan syndrome 2	605275	AR			
**MAP2K1**	176872	Noonan syndrome and related disorders	H00523		Ras pathway	map04014	Activation
					Vascular smooth muscle contraction	map04270	Activation
**MAP2K2**	601263	Noonan syndrome and related disorders	H00523		Ras pathway	map04014	Activation
					Vascular smooth muscle contraction	map04270	Activation
**MAP4K4**	604666	Lymphatic vascular development	[156]		Ras pathway	[157]	Inactivation
**MET**	164860	Lymphedema	[27]		Ras pathway	map04014	Activation
					PI3K/AKT pathway	map04151	Activation
**NFATC1**	600489	Lymphatic valve development	[158]		Transcription factors	[158]	Activation
**NOTCH1**	190198	Lymphatic vascular development	[77]		Transcription factors	[77]	Inactivation
**NPPA**	108780	Lymphatic vessels contraction and permeability	[159]		Vascular smooth muscle contraction	map04270	Activation
**NPPB**	600295	Lymphatic vessels contraction and permeability	[159]		Vascular smooth muscle contraction	map04270	Activation
**NR2F2**	107773	Lymphatic vascular development	[77]		Transcription factors	[77]	Activation
**NRP1**	602069	Lymphedema	[160]		Rho and cytoskeletal remodelling pathway	[127]	Activation
**NRP2**	602070	Lymphedema	[160]		Rho and cytoskeletal remodelling pathway	[127]	Activation
**PDPN**	608863	Lymphedema	[161]		Rho and cytoskeletal remodelling pathway	[60]	Activation
**PLCG2**	600220	Lymphatic vascular development	[162]		Ras pathway	map04014	Activation
**PLXNA1**	601055	Lymphatic valve development	[163]		PI3K/AKT pathway	[164]	Inactivation
					Rho and cytoskeletal remodelling pathway	[164]	Inactivation
**PPP1CB**	600590	Noonan syndrome-like disorder with loose anagen hair 2	617506	AD	Hippo pathway	hsa04390	Activation
					Ras pathway	[165]	Inactivation
					Vascular smooth muscle contraction	map04270	Inactivation
**PROX1**	601546	Lymphedema	[74]		Ras pathway	[38]	Activation

**RAF1**	164760	Noonan syndrome 5	611553	AD	Ras pathway	map04014	Activation
					Vascular smooth muscle contraction	map04270	Activation
**RAMP2**	605154	Lymphatic vascular development	[123]		Ras pathway	[124]	Activation
					PI3K/AKT pathway	[124]	Activation
					Vascular smooth muscle contraction	map04270	Activation
**RELN**	600514	Lissencephaly 2 (Norman-Roberts type)	257320	AR	PI3K/AKT pathway	map04151	Activation
**RORC**	602943	Lymphedema	[83]		Transcription factors	[83]	Activation
**S1PR1**	601974	Lymphatic vascular development	[61]		Ras pathway	[166]	Activation
					PI3K/AKT pathway	[166]	Activation
					NFkB pathway	[166]	Activation
**S1PR2**	605111	Lymphatic vessels contraction	[167]		Ras pathway	[166]	Activation
					Rho and cytoskeletal remodelling pathway	[166]	Activation
					PI3K/AKT pathway	[166]	Activation
					NFkB pathway	[166]	Activation
**S1PR3**	601965	Lymphatic vascular development	[168]		Rho and cytoskeletal remodelling pathway	[166]	Activation
**S1PR4**	603751	Lymphatic vascular development	[152]		Ras pathway	[166]	Activation
**S1PR5**	605146	Lymphatic vascular development	[152]		Ras pathway	[166]	Activation
**SDC4**	600017	Lymphatic vascular development	[169]		Planar cell polarity	[170]	Activation
**SEMA3A**	603961	Lymphedema	[171]		Rho and cytoskeletal remodelling pathway	[172]	Activation
**SMARCA4**	603254	Lymphatic vascular development	[173]		Transcription factors	[174]	Activation
**SOS2**	601247	Noonan syndrome 9	616559	AD	Ras pathway	map04014	Activation
**SOX17**	610928	Lymphatic vascular development	[175]		Transcription factors	[175]	Activation
**SPRED1**	609291	Lymphatic vascular development	[38]		Ras pathway	[38]	Inactivation
**SPRED2**	609292	Noonan syndrome-like phenotype	[176]		Ras pathway	[38]	Inactivation
**SVEP1**	611691	Lymphedema	[104]		Cell adhesion	[104]	Inactivation
					PI3K/AKT pathway	[177]	Activation
**SYK**	600085	Lymphedema	[137]		PI3K/AKT pathway	map04151	Activation
**PECAM1**	173445	Lymphatic valve development	[100]		Mechanotrasduction	[100]	Activation
**TACR1**	162323	Lymphedema	[178]		Ras pathway	[179]	Activation
**TIE1**	600222	Lymphatic malformation 11	619401	AD	PI3K/AKT pathway	[79]	Inactivation
					Ras pathway	[180]	Inactivation
**VANGL2**	600533	Lymphedema	[89]		Planar cell polarity	[65]	Activation
					Rho and cytoskeletal remodelling pathway	[65]	Activation
**VCAM1**	192225	Lymphedema	[137]		NFkB pathway	map04064	Activation
